# Supplementary material for: MetaRibo-Seq measures translation in microbiomes
Source: Nat Commun. 2020 Jun 29;11:3268. doi: 10.1038/s41467-020-17081-z (PMC7324362; doi:10.1038/s41467-020-17081-z)
Supplement: Supplementary file 10 — Supplementary Data 7 [file 41467_2020_17081_MOESM10_ESM.zip › File2/Confidence_High_Taxonomy/45014_out.krona.html]

Javascript must be enabled to view this page.

members
magnitude
magnitudeUnassigned
count
unassigned
taxon
rank

45014\_out

2

2
2
superkingdom

phylum
2
1239

2
186801
class

2
186802
order

family
31979
2

2
1485
genus

species

SRS075078\_contig\_number\_contig-100\_2583.141580SRS098644\_contig\_number\_15879
2
1262777
